# Chronic anemia: The effects on the connectivity of white matter

**DOI:** 10.3389/fneur.2022.894742

**Published:** 2022-07-26

**Authors:** Clio González-Zacarías, Soyoung Choi, Chau Vu, Botian Xu, Jian Shen, Anand A. Joshi, Richard M. Leahy, John C. Wood

**Affiliations:** ^1^Neuroscience Graduate Program, University of Southern California, Los Angeles, CA, United States; ^2^Signal and Image Processing Institute, University of Southern California, Los Angeles, CA, United States; ^3^Department of Pediatrics and Radiology, Children's Hospital Los Angeles, Los Angeles, CA, United States; ^4^Biomedical Engineering, University of Southern California, Los Angeles, CA, United States

**Keywords:** chronic anemia, diffusion MRI, tensor analysis, sickle cell disease (SCD), thalassemia, white matter (WM)

## Abstract

Chronic anemia is commonly observed in patients with hemoglobinopathies, mainly represented by disorders of altered hemoglobin (Hb) structure (sickle cell disease, SCD) and impaired Hb synthesis (e.g. thalassemia syndromes, non-SCD anemia). Both hemoglobinopathies have been associated with white matter (WM) alterations. Novel structural MRI research in our laboratory demonstrated that WM volume was diffusely lower in deep, watershed areas proportional to anemia severity. Furthermore, diffusion tensor imaging analysis has provided evidence that WM microstructure is disrupted proportionally to Hb level and oxygen saturation. SCD patients have been widely studied and demonstrate lower fractional anisotropy (FA) in the corticospinal tract and cerebellum across the internal capsule and corpus callosum. In the present study, we compared 19 SCD and 15 non-SCD anemia patients with a wide range of Hb values allowing the characterization of the effects of chronic anemia in isolation of sickle Hb. We performed a tensor analysis to quantify FA changes in WM connectivity in chronic anemic patients. We calculated the volumetric mean of FA along the pathway of tracks connecting two regions of interest defined by BrainSuite's BCI-DNI atlas. In general, we found lower FA values in anemic patients; indicating the loss of coherence in the main diffusion direction that potentially indicates WM injury. We saw a positive correlation between FA and hemoglobin in these same regions, suggesting that decreased WM microstructural integrity FA is highly driven by chronic hypoxia. The only connection that did not follow this pattern was the connectivity within the left middle-inferior temporal gyrus. Interestingly, more reductions in FA were observed in non-SCD patients (mainly along with intrahemispheric WM bundles and watershed areas) than the SCD patients (mainly interhemispheric).

## Introduction

Chronic anemia (CA) is a condition in which the number of erythrocytes or hemoglobin (Hb) concentration is lower than expected and incapable of meeting physiological needs (1). Worldwide, the prevalence of anemia is very high, affecting around 1.93 billion people and causing more significant disability than asthma, diabetes, and cardiovascular disease combined (2). Tissue oxygen consumption is heterogeneous and organ-specific. The brain is one of the organs with higher metabolic demand that receives preferential blood flow under acute circumstances (3). As a result, neurons are specifically sensitive to hypoxia (4). CA causes reduced oxygenation in the brain, leading to hypoxia, neuroinflammation, and white matter (WM) remodeling (5).

CA is a standard clinical feature seen in patients with hemoglobinopathies (6), mainly represented by qualitative disorders in Hb structure (e.g., sickle cell disease, SCD) and quantitative disorders of Hb synthesis (e.g., thalassemia syndromes, non-SCD) (7). Hemoglobinopathies have been associated with gray matter (GM) (8–11) and WM alterations (12–17), cerebral vasculopathies (18–20), and changes in cerebral blood flow (21–26), thus serving as a model for the cerebral changes caused by CA (15, 17).

Studying SCD and non-SCD patients with a wide range of Hb values and genetic predisposition to anemia simultaneously allows the characterization of the effects of CA in isolation from sickle Hb (17, 22). In this context, novel structural magnetic resonance imaging (MRI) research done in our laboratory, where SCD and non-SCD patients were simultaneously analyzed, demonstrated that WM volume was diffusely lower in deep, watershed areas proportional to anemia severity regardless of Hb genotype (15). This relationship between anemia and WM volume was confirmed in a repeated analysis with a restricted population consisting of patients with beta-thalassemia (27).

We hypothesized that CA causes similar damaging effects and changes in structural connectivity of WM in patients with hemoglobinopathies. Furthermore, the damage is driven by hyperemia and not by the intrinsic pathophysiology of these hemoglobinopathies. To characterize alterations in the WM, we performed a diffusion tensor imaging (DTI) analysis and quantified the average fractional anisotropy (FA, overall directionality of water diffusion) along the pathways connecting every pair of regions of interest (ROIs) defined by an anatomical atlas. This approach covers all the WM bundles in the brain and not merely the main association fascicles (28).

Diffusion MRI (dMRI) is used to study in-vivo WM microstructure and allows quantitative characterization of healthy and diseased tissue. The most widely used dMRI technique is DTI, despite various limitations. Its derived metrics like FA are potential biomarkers of brain abnormalities in patients with neurodegenerative diseases. For example, DTI analysis already provided evidence of the relationship between WM microstructure and markers of anemia severity, such as oxygen saturation level and Hb value (13). Additionally, SCD patients have shown lower FA in the corticospinal tract and cerebellum (13), across the internal capsule (14), the corpus callosum (14, 16, 29, 30), and in the deep WM (30). Decrement of FA was also observed in major WM tracts in CA patients, regardless of the anemia subtype, and correlated significantly with the neurocognitive decline observed in the CA population (29).

## Materials and methods

### Participation criteria

All participants in this study were part of a larger project on Sickle Cell Disease at Children's Hospital Los Angeles that its Institutional Review Board approved. Each participant was recruited with informed consent. We collected MRI data and blood tests on patients, including non-SCD, SCD, and healthy controls matched by sex and age. The accepted age range was between 10 to 50 years old. Eligibility criteria included patients with SCD diagnosis (Hb SS, Hb SC, Hb Sβ^0^, and Hb Sβ^+^genotypes), patients with chronic anemia diagnosis (beta-thalassemia major, beta-thalassemia intermedia, hemoglobin H-constant spring, congenital dyserythropoietic anemia, spherocytosis anemia, and autoimmune hemolytic anemia) and healthy controls (mainly recruited from family members of SCD patients to match race and ethnicity between groups). The exclusion criteria disqualified those patients with previous overt stroke, acute chest syndrome, pain crisis hospitalization (within one month), and pregnant candidates. Similarly, individuals with prior history of neurologic insults, developmental delay, or chronic medical conditions that require regular medical care or medications and pregnant candidates would be ineligible as healthy controls.

We followed the standard guidelines and regulations for MRI safety and exclusion criteria. On the same day, each participant completed the MRI examination without sedating medications, and we collected vital signs and blood samples.

### Laboratory markers

To account for the similarities and differences in CA pathophysiology, all participants enrolled in our study underwent a thorough examination of their blood samples. Complete blood count, reticulocyte total, and quantitative Hb electrophoresis percentages of Hb S, Hb A, Hb A2, hemoglobin F, etc.) were analyzed in the clinical laboratory. Additional surrogates for hemolysis like lactate dehydrogenase (LDH) and plasma-free Hb levels were also quantified (Table 1).

**Table 1:** Demographics.

	**CTL**	**non-SCD**	**SCD**	**CTL vs. non-SCD^†^**	**CTL vs. SCD^†^**	**non-SCD vs. SCD^†^**
N	23	15	19	-	-	-
Sex (F:M)	14:9	8:7	10:9	-	-	-
Age	21.3 ± 5.9	22.4 ± 4.8	21.8 ± 8.3	0.88	0.97	0.96
Transfused	0	9	5	-	-	-
Hemoglobin (g/dL)	13.2 ± 1.2	10.3 ± 1.7	10.2 ± 2.1	≤0.01	≤0.01	0.96
Hematocrit (%)	39.8 ± 3.6	32.2 ± 5.82	28.7 ± 5.3	≤0.01	≤0.01	0.10
White blood cell count (x10^3^)	5.6 ± 1.7	7.0 ± 3.1	9.3 ± 4.3	0.33	≤0.01	0.11
Reticulocytes (%)	1.2 ± 0.5	3.1 ± 3.1	7.8 ± 3.5	0.07	≤0.01	≤0.01
Plasma-free hemoglobin	6.7 ± 5.2	22.3 ± 23.8	21.8 ± 19.6	0.02	0.02	0.99
Lactose dehydrogenase	519.5 ± 75.2	632.2 ± 361.5	1008.5 ± 573.7	0.64	≤0.01	≤0.01
Absolute neutrophil count	3.2 ± 1.6	4.2 ± 1.8	5.4 ± 3.6	0.41	≤0.01	0.37
Heart rate (min^−1^)	70.5 ± 20.4	79.0 ± 13.4	81.7 ± 13.9	0.28	0.08	0.88
Systolic blood preassure (mmHg)	116.1 ± 9.2	113.9 ± 9.1	115.5 ± 12.6	0.79	0.98	0.89
Dyastolic blood preassure (mmHg)	66.1 ± 9.5	60.3 ± 9.3	63.7 ± 7.3	0.13	0.65	0.51
O_2_ saturation (%)	99.5 ± 0.9	98.3 ± 2.9	97.8 ± 1.6	0.17	≤0.01	0.66
Hemoglobin A (%)	82.2 ± 17.9	90.7 ± 7.9	23.4 ± 32.4	-	-	-
Hemoglobin F (%)	0.7 ± 2.4	2.9 ± 4.1	8.8 ± 9.5	-	-	-
Hemoglobin S (%)	14.1 ± 18.1	0.0 ± 0.0	50.9 ± 26.0	-	-	-

*Mean and standard deviation of demographic information and selected complete blood count measurements*.

*CTL, control; non-SCD, non sickle cell disease; SCD, sickle cell disease*.

† *Group comparison using one-way analysis of variance (ANOVA) result with Tukey-Kramer test for multiple comparisons. Statistically significant values (p ≤ 0.05) are color-coded as follows: green color denotes the comparison between CTL and non-SCD groups, red compares CTL and SCD, and blue color non-SCD with SCD patients*.

### Image acquisition

The MRI data were acquired on a 3T Philips Achieva scanner using an 8-channel head coil for each participant. The structural 3D T1-weighted (T1-w) sequence specification was TR/TE = 8.3/3.8 ms, SENSE = 2, and isotropic voxel size of 1 mm^3^. In addition, a single-shell dMRI sequence was acquired with TR/TE = 6,700/86 ms; isotropic voxel size of 2.5 mm^3^; 30 diffusion-encoding directions at b-value = 1,000 s/mm^2^ and one b-value = 0 s/mm^2^ using a single-shot echo-planar imaging sequence.

### Post-processing

The Brain extraction and parcellation from the T1-weighted (T1-w) images were processed with BrainSuite (brainsuite.org, v19b). Specifically, BrainSuite's Cortical Surface Extraction (CSE) tool was used to perform skull stripping (31), tissue classification, including partial volume fraction of voxels identified as WM, GM, and CSF (32), topological corrections (33), and delineation of the inner/outer cortex. In addition, BrainSuite's Surface Volume Registration (SVReg) tool (34, 35) performed anatomical co-registration to the BCI-DNI anatomical atlas (35), and brain segmentation.

The dMRI data were corrected for localized geometric distortions to enable accurate multi-modal analysis. Each subject's motion and eddy current-induced distortions were corrected with FSL's eddy module (36, 37). Using BrainSuite's Diffusion Pipeline (BDP), we registered the dMRI to the T1-w data, followed by susceptibility-distortion correction based on the inverse contrast normalization (38).

### Diffusion modeling

Using the well-known tensor equation, we calculated the fractional anisotropy maps in BDP. To render more accurate tractography in the WM, we also computed in BDP the orientation density functions (ODFs). In particular, the ensemble average propagator response function optimized (ERFO) uses machine learning and linear estimation theory to optimize ODF accuracy for arbitrary q-space sampling schemes. It has shown advantages over other methods (39). Furthermore, ERFO can model single-shell (and multi-shell) data and has the capacity of rendering crossing fibers with the most negligible false positives (40).

**Figure 1:**
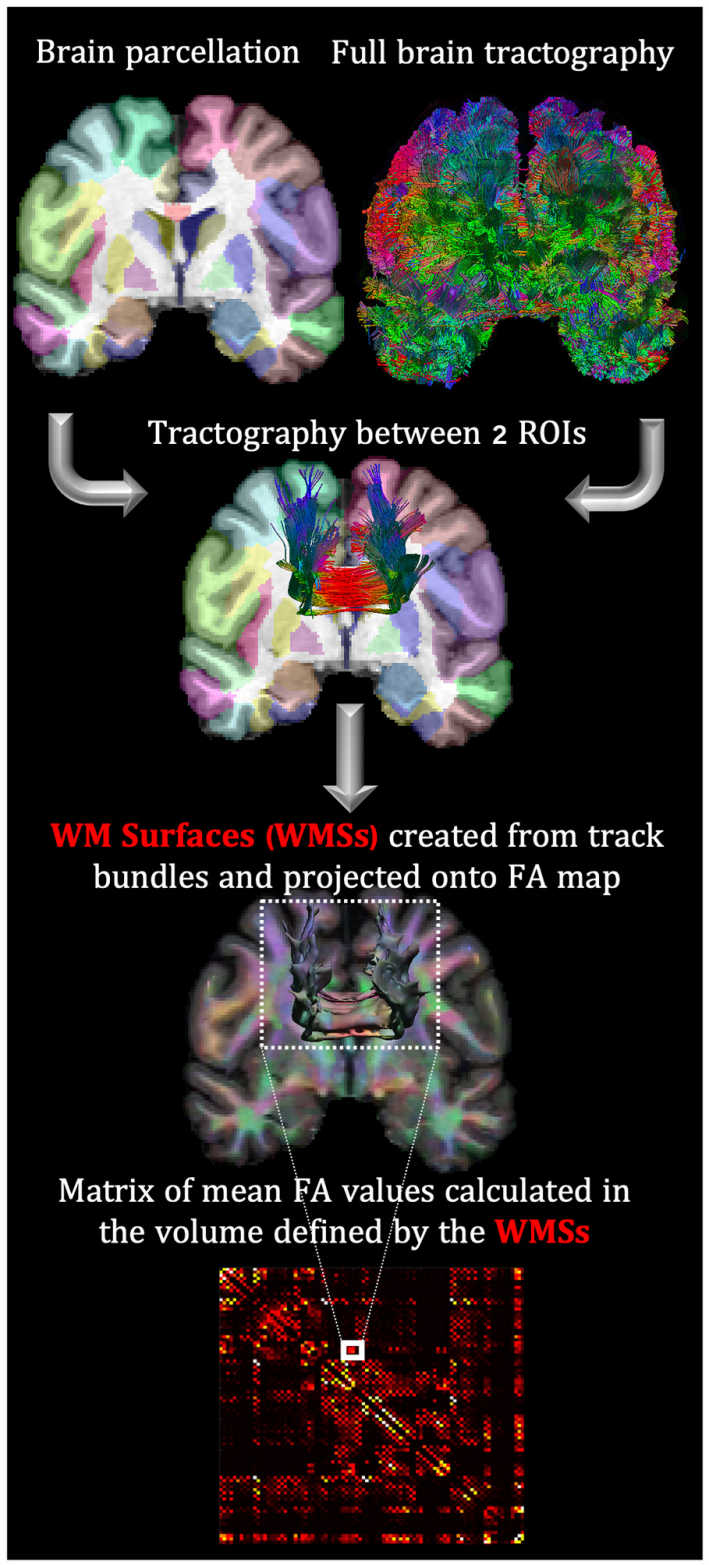
FA analysis in WMS based on the coregistration of the parceled T1-w with dMRI maps. This coregistration allows mapping the connectivity tracks between two ROIs to the FA map. The exact process is repeated to create a connectivity matrix for each subject.

Whole-brain deterministic fiber tracking, based on quantitative anisotropy (41), and visualization were performed with the DSI Studio tractography package (http://dsi-studio.labsolver.org). The sections of tracks entering cortical GM or subcortical regions were excluded to avoid partial volume effects. Afterward, detail connectivity analysis of fiber bundles connecting two ROIs (previously labeled on the T1-w structural images) was implemented with the TractConnect Matlab package (https://neuroimage.usc.edu/neuro/Resources/TractConnect). Specifically, TractConnect uses filtered tracks connecting two ROIs to define a volumetric white matter surface (WMS) and projects it into the FA maps (Figure 1). Then FA values within WMSs were averaged.

For each individual, the average FA values were used as the elements of the connectivity matrix. 88 ROIs from the BCI-DNI atlas were used: 66 cortical regions, 14 subcortical, corpora quadrigemina, mammillary bodies, brainstem, and cerebellum.

Overall, this modeling method offers higher sensitivity and specificity to detect not only regional differences in WM microstructure (like voxel-wise analysis would do) but along the connectivity pathways, and it is robust to some of the commonly criticized featured of DTI ((42, 43)): the inability to render crossing fibers and to define connectivity between ROIs by streamlining counting. The first was overcome by using ERFO to model diffusion ODFs and the latter by defining the WMSs and characterizing these “connections” with the mean FA value.

### Statistical analysis

For each element of the connectivity matrix (upper triangular part), Figure 1, the FA differences between groups were modeled using multiple linear regression analysis after controlling for logarithm of age (log-age), sex, and group. The logarithm of age was used because brain maturational effects are nonlinear with age in adolescents and young adults (44, 45). Finally, the results were also corrected for multiple comparisons using the False Discovery Rate (FDR) to adjust the correspondent p-values (46) with a 20% acceptance rate.

A similar analysis was performed using a permutation analysis using Manly's method (47, 48), which was also FDR corrected (49) with the same threshold. Given the complexity of the data, it was not possible to guarantee all the assumptions of linear modeling. Consequently, we also chose to model the WMSs using nonparametric permutation analysis. Overlapping between the two methods provided an additional confidence level in our results.

For completeness, we tested the possible contribution of changes in FA caused by monthly transfusions and LDH values in patients. For this, we only ran a multiple linear regression analysis controlling also for log-age, sex, and group. All the statistical analyses were calculated using the R statistical package (50).

## Results

### Demographics

In this analysis we considered 19 clinically asymptomatic SCD patients (age = 22.4 ± 7.8 years; Hb = 10.1 ± 2.1 g/dL; F = 9 patients), 15 non-SCD anemic patients (age = 22.4 ± 4.8 years; Hb = 10 ± 2.8 g/dL; F = 8 patients) and 23 control subjects (age = 21.3 ± 6 years; Hb = 13.3 ± 1.2 g/dL; F = 14 individuals). The age range for all the participants was 11.2 to 35.8 years. All demographics are reported in Table 1.

The breakdown of the race (and ethnicity) for control subjects was 17 African-American (non-Hispanic) and 5 White (Hispanic) individuals. SCD patients included 17 African-American (non-Hispanic) and 2 White (Hispanic) patients. The non-SCD group consisted of 8 Asian (non-Hispanic), 5 White (non-Hispanic), and 2 White (Hispanic) patients.

For the SCD group, the genotypes were 12 Hb SS and 7 Hb SC patients. Because of the specific matches between control and SCD, 9 of the control subjects were identified with sickle cell trait having hemoglobin AS (Hb AS). Previous work in our laboratory suggests that the Hb AS subtype does not alter normal cerebral blood flow (CBF) regulation and balance of oxygen supply and demand Field (22), making Hb AS carriers good candidates for control subjects.

The specific anemias in the non-SCD group consisted of 7 patients with beta-thalassemia major, 3 beta-thalassemia intermedia, 2 hemoglobin H-constant spring, 1 congenital dyserythropoietic anemia, 1 spherocytosis anemia and 1 autoimmune hemolytic anemia.

Of the CA patients, 8 non-SCD (7 beta-thalassemia major and 1 congenital dyserythropoietic anemia) and 5 SCD (Hb SS patients) were on monthly transfusions. The rest of the non-transfused SS patients were prescribed hydroxyurea and had a mean hemoglobin F fraction of 18%. One patient with SC was also taking hydroxyurea. At Children's Hospital Los Angeles, it is recommended to treat all pediatric patients, of all SCD genotypes, nine months and older with hydroxyurea unless they have been placed on chronic transfusion (51, 52), as indicated by NIH guidelines (53). Furthermore, as of 2,000, all SCD patients at our facility have received access to the transcranial Doppler screening (51–54).

### Laboratory comparisons

Laboratory and clinical markers are shown in Table 1. Hemoglobin (*p* = 0.96) and hematocrit (*p* = 0.10) levels were not statistically different between CA groups, but both had statistically significant lower values compared with healthy control (non-SCD, SCD: *p* ≤ 0.01). Furthermore, the SCD population showed significantly higher levels of reticulocytes (*p* ≤ 0.01) and LDH (*p* ≤ 0.01) compared to both non-SCD and control, suggesting increased intravascular hemolysis. However, plasma-free Hb was not different between CA types (*p* = 0.99). SCD patients had mildly increased white cell counts with respect to control subjects (*p* ≤ 0.01) but not relative to the non-SCD anemic patients (*p* = 0.11). In the case of Hb electrophoresis, Hb S was highest for SCD patients. Still, our control also exhibited a smaller percentage of Hb S because of the inclusion of sickle trait subjects. SCD patients demonstrated the highest hemoglobin F (Hb F) concentration, with intermediate levels observed in non-SCD patients. Most control subjects had no Hb F, but one subject had 11.7% Hb F.

### White matter connectivity

Overall, no statistically significant WMSs were found when comparing SCD with non-SCD patients. 10 WMSs in CTL vs. non-SCD and 5 WMSs in CTL vs. SCD analysis showed significant differences in both multiple linear regression and permutation analysis (Table 2). ^*^FA indicates the group mean FA after controlling for log-age and sex using the multiple linear regression. The point-biserial correlation coefficient, $r_{{}^{*}FA,Gr}$, shows the direction and strength of the relationship between ^*^FA and the status of being anemic or not. A negative $r_{{}^{*}FA,Gr}$ value depicts higher ^*^FA in control individuals than CA patients. All WMSs reported in Table 2 showed this behavior except for one (left middle and inferior temporal gyrus) in healthy controls vs. non-SCD comparison. The ^*^FA unpaired two-samples t-test statistic is also displayed for completeness, which agrees with the multiple linear regression analysis.

**Table 2:** Results for ^*^ FA.

**ROI-1**	**ROI-2**	CTL ^*^FA	CA ^*^FA	r_^*^FA, Hg_^†^	**T-statictic^†^**	r_^*^FA, Hg_^¥^	p_^*^A, Hg_^¥^
**CTL vs. non-SCD**							
R. caudate nucleus	R. middle frontal gyrus	0.37	−0.57	−0.46	t(36) = 3.1 *p ≤* 0.01	0.29	≤0.01
R. thalamus	R. middle frontal gyrus	0.49	−0.70	−0.59	t(30) = 3.9 *p ≤* 0.01	0.41	≤0.01
R. thalamus	R. amygdala	0.47	−0.65	−0.55	t(34) = 3.8 *p ≤* 0.01	0.51	≤0.01
L. thalamus	L. gyrus rectus	0.40	−0.63	−0.50	t(34) = 3.4 *p ≤* 0.01	0.37	≤0.01
L. thalamus	L. parahipppocampal gyrus	0.40	−0.56	−0.48	t(34) = 3.2 *p ≤* 0.01	0.32	0.06
R. superior frontal gyrus	R. cingulate gyrus	0.46	−0.73	−0.58	t(34) = 4.2 *p ≤* 0.01	0.41	≤0.01
R. transvers frontal gyrus	R. subcallosal gyrus	0.49	−0.68	−0.59	t(31) = 4.0 *p ≤* 0.01	0.37	0.03
R. cingulate gyrus	L. cingulate gyrus	0.52	−0.72	−0.61	t(34) = 4.5 *p ≤* 0.01	0.53	≤0.01
L. cingulate gyrus	L. pre-cuneus	0.45	−0.82	−0.61	t(32) = 4.3 *p ≤* 0.01	0.39	0.02
L. middle temporal gyrus	L. inferior temporal gyrus	−0.40	0.62	0.50	t(34) = 3.3 *p ≤* 0.01	−0.25	≤0.01
**CTL vs. SCD**							
L. thalamus	L. parahipppocampal gyrus	0.50	−0.55	−0.54	t(38) = 3.9 *p ≤* 0.01	0.46	≤0.01
R. gyrus rectus	L. middle orbito-frontal gyrus	0.63	−0.73	−0.68	t(37) = 5.6 *p ≤* 0.01	0.35	0.03
R. middle orbito-frontal gyrus	L. middle orbito-frontal gyrus	0.48	−0.59	−0.54	t(36) = 3.8 *p ≤* 0.01	0.28	0.09
L. middle orbito-frontal gyrus	R. subcallosal gyrus	0.54	−0.63	−0.58	t(39) = 4.5 *p ≤* 0.01	0.42	≤0.01
L. middle orbito-frontal gyrus	L. subcallosal gyrus	0.58	−0.71	−0.64	t(38) = 5.1 *p ≤* 0.01	0.27	0.1

*Connectivity between ROI-1 and ROI-2 that was statistically significant after FDR correction in multilinear and permutation models controlling for the group, sex, and age (log-transformed). The upper and lower sections of the table show the statistics for FA when comparing healthy controls (CTL) with non-SCD and SCD patients. No conections were statistically significat when comparing non-SCD with SCD patients*.

* *Mean group FA controlled for sex and age (log-transformed) along the volumetric white matter surface connecting these ROIs. Standardized (unitless) values are shown*.

† Point-biserial correlation coefficient and results of the unpaired two-samples t-test on the ^*^FA values between groups.

¥ Pearson correlation of ^*^FA with Hb and the correspondent p-value is also displayed.

When Hb was included in the mathematical models as a covariate, all the ^*^FA differences reported in Table 2 were no longer statistically significant. This suggests that many of the effects reported are driven by the Hb differences between healthy controls and CA patients. To further study the relationship between ^*^FA and Hb, we calculated the Pearson correlation coefficient, $r_{{}^{*}FA,Hb}$, and their respective p-value, $p_{{}^{*}FA,Hg}$, for the WMSs reported in Table 2. ^*^FA was significantly correlated with Hb levels in 8 out of the 10 WMSs in the population consisting of control and non-SCD analysis and in 3 out of the 5 WMSs in the control and SCD population. Consequently, by calculating $r_{{}^{*}FA,Hb}^{2}$, the proportion of variance in ^*^FA explained by Hb, we observed that in control and non-SCD for the WMSs reported in Table 2, Hb accounts for up to 26% (right thalamus and right amygdala) of the variance in ^*^FA, and up to 21% (left thalamus and left parahippocampal gyrus) in the case of controls with SCD patients.

**Figure 2:**
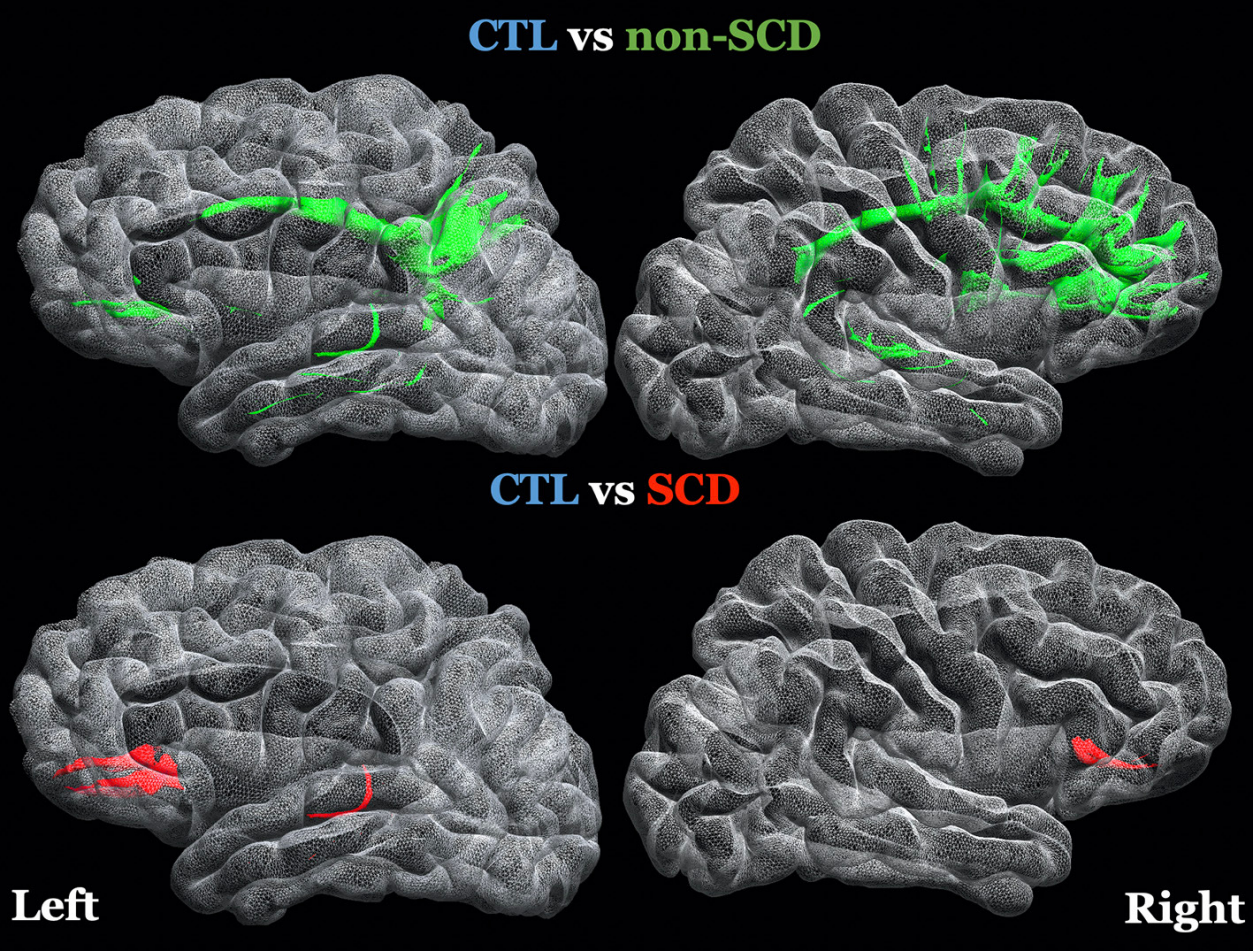
3D-rendering of left and right hemispheres of a representative subject, the white matter surfaces (WMSs) where FA was controlled for age (log transformed), sex and group and it was statistically significant in both statistical models (linear regression and permutation analysis) after FDR correction. The specific regions of interest are listed on Table 2. Top Row: green WMSs, comparison of healthy controls (CTL) with non-sickle cell anemia (non-SCD). Bottom row: red WMSs, comparison of CTL with sickle cell anemia (SCD).

The spatial locations of the WMSs listed in Table 2 are 3D-rendered in the left and right hemispheres of a representative subject (Figure 2). In these same WMSs, we saw a positive correlation of ^*^FA with Hb. Significant results were bilateral and generally symmetrical across hemispheres. Interestingly, more WMSs survived for the non-SCD (mainly intrahemispheric and along with watershed areas) than for the SCD (mainly interhemispheric) group compared with healthy controls.

Table 3 shows the results of the multiple linear regression when adding in the model transfusion status. For non-SCD patients, 4 out of 10 WMSs displayed in Table 2 were still statistically significant, and for SCD patients, all the WMSs reported in Table 2 still appeared. When including LDH as a marker of hemolysis in the mathematical model, 6 out of 10 WMSs were statistically significant for non-SCD and 4 of 5 WMSs for SCD patients.

**Table 3:** Results of FA when controlling for transfusion status and lactose dehydrogenase (LDH).

**ROI-1**	**ROI-2**	**T-statictic**	
		**Transfusion^♦^**	**LDH^♢^**
**CTL vs non-SCD**			
R. caudate nucleus	R. middle frontal gyrus	-	-
R. thalamus	R. middle frontal gyrus	t(30) = 3.0 *p ≤* 0.01	t(30) = 3.4 *p ≤* 0.01
R. thalamus	R. amygdala	-	t(34) = 3.5 *p ≤* 0.01
L. thalamus	L. gyrus rectus	-	-
L. thalamus	L. parahipppocampal gyrus	-	-
R. superior frontal gyrus	R. cingulate gyrus	t(34) = 2.9 *p ≤* 0.01	t(34) = 3.5 *p ≤* 0.01
R. transvers frontal gyrus	R. subcallosal gyrus	-	t(31) = 4.1 *p ≤* 0.01
R. cingulate gyrus	L. cingulate gyrus	t(34) = 2.7 *p ≤* 0.01	t(34) = 4.0 *p ≤* 0.01
L. cingulate gyrus	L. pre-cuneus	t(32) = 2.3 *p ≤* 0.01	t(32) = 3.8 *p ≤* 0.01
L. middle temporal gyrus	L. inferior temporal gyrus	-	t(34) = −3.2 *p ≤* 0.01
**CTL vs SCD**			
L. thalamus	L. parahipppocampal gyrus	t(38) = 3.9 *p ≤* 0.01	-
R. gyrus rectus	L. middle orbito-frontal gyrus	t(37) = 5.6 *p ≤* 0.01	t(37) = 4.1 *p ≤* 0.01
R. middle orbito-frontal gyrus	L. middle orbito-frontal gyrus	t(36) = 3.8 *p ≤* 0.01	t(36) = 2.8 *p ≤* 0.01
L. middle orbito-frontal gyrus	R. subcallosal gyrus	t(39) = 4.5 *p ≤* 0.01	t(39) = 3.2 *p ≤* 0.01
L. middle orbito-frontal gyrus	L. subcallosal gyrus	t(38) = 5.1 *p ≤* 0.01	t(38) = 4.1 *p ≤* 0.01

*Connectivity between ROI-1 and ROI-2 that was statistically significant after FDR correction in the multilinear model. For consistency same ROIs are displayed as in Table 1. The upper and lower sections of the table show the statistics for FA when comparing healthy controls (CTL) with non-SCD and SCD patients. Similar to Table 2, no conections were statistically significat when comparing non-SCD with SCD patients*.

♦ *Unpaired two-sample t-test on FA values controlled for the group, sex, age (log-transformed), and transfusion status*.

♢ *Unpaired two-sample t-test on FA values controlled for the group, sex, age (log-transformed), and LDH values*.

## Discussion

We observed potential microstructural differences along WMSs in both groups of patients with CA with and without SCD compared to controls. These results predominantly showed lower FA values in CA patients, indicating the loss of coherence in the main diffusion direction, which could indicate WM injury. Lower FA was highly associated with decreasing Hb levels revealing that the decreased microstructural integrity found in CA patients is highly driven by chronic hypoxia.

Previous work in CA has shown that the whole brain increases cerebral blood flow (CBF) to compensate for the loss of oxygen-carrying capacity (21, 24, 55–57). This offset in CBF preserves total resting oxygen delivery to the whole brain (21, 22, 55, 58), such that the correspondent oxygen extraction fraction (OEF) from the cerebral cortex seems to be normal or even reduced (26, 59–62). Although resting oxygen delivery is preserved in the cortex, the cerebral vascular reserve is diminished, proportional to the resting hyperemia (23, 25), potentially leaving the brain vulnerable to acute insults such as nighttime hypoxia, acute anemia, and fever. However, while there have been some reports of reduced cortical and subcortical GM volumes (9, 63, 64) in patients not receiving chronic transfusion or hydroxyurea treatment, total GM volume appears preserved (65) in clinically asymptomatic SCD patients (adolescents and young adults) treated with either therapy suggesting that cortical volume loss may not manifest until later in life.

However, regions of deep WM do not seem to have the same metabolic stability during periods of ischemic stress. OEF is increased in the deep watershed areas (56), colocalizing with WM injury patterns (18, 58, 66). Chai et al. (58) showed that independently of disease state, CBF and oxygen delivery to regions of deep WM and border zone regions are considerably smaller than those measured elsewhere within the WM and GM. Furthermore, Wang et al. (30) showed that elevated CBF can be associated with normal-appearing (i.e., infarct-free) WM disruption. Inadequate resting oxygen delivery in the WM is further compounded by blunted cerebrovascular reserve (25).

Thus, chronic hypoperfusion plays a role in the development of the entire WM damage phenotype, including hyperintensities on the T2 FLAIR (17, 18, 58, 66, 67), reduced WM volume (15, 27, 68) or changes in diffusion metrics (12–14, 16, 29, 30).

Based on this consideration, the damage observed in non-SCD patients compared with controls followed an intuitive pattern located primarily in the frontal-parietal WM watershed areas (Table 2). Watershed areas are regions in the brain that sit in-between major cerebral arterial territories and are the most susceptible to hypoxic-ischemic damage when a supply-demand mismatch occurs in the cerebrovascular supply (18, 58, 69).

While most of the affected connections were unilateral, WMSs observed with lower FA in non-SCD patients appeared similarly distributed between the two hemispheres (Figure 2). These results are consistent with the spatial patterns of lower WM volume associated with the severity of anemia diffusely across frontal, parietal lobes, and temporal lobes especially in these watershed areas (15, 68).

Given that most of the WMSs survived when controlling for a hemolysis marker (Table 3), the results are also aligned to a model of global hypoxia that will usually cause diffuse, bilateral brain injury as seen in patients in drowning accidents, cardiac arrest, or bilateral carotid stenosis, in contrast to more localized and asymmetric injury patterns caused by embolic stroke (70). Therefore, we suggest that the injury pattern in WM microstructure of non-SCD patients can indicate global chronic hypoxia driven by anemia's effect on the brain's hemodynamics. A possible explanation is that the vascular architecture providing blood perfusion to WM areas is the long-penetrating medullary arteries with poor collateralization. Consequently, WM is especially vulnerable to hyperintensities development under focal ischemic events or periods of acute stress (71).

For SCD patients and healthy controls, three out of five connections crossed to the contralateral side. Interhemispheric involvement is consistent with previous results from our laboratory showing lower FA in the corpus callosum in CA patients (higher burden on SCD) (29). There are similar observations on SCD in studies performed in Tanzania (16), the United Kingdom (14), and the United States (30). Kawadler et al. (13) also showed associations between microstructural properties in the corpus callosum with daytime oxygen saturation and Hb levels in SCD patients, indicating that hypoxia related at least in part to low hemoglobin in SCD patients drives the WM injury patterns.

Previous DTI studies in SCD patients have also reported widespread FA decrease in the WM (12–14, 30). Surprisingly, we did not observe this extent of systematic FA derangements. This difference possibly reflects the variability in disease expression in our SCD cohort compared to previous reports; 7 subjects had SC genotype, and 5 of the 12 SS patients were receiving chronic transfusions. While SC and chronically transfused patients develop WM hyperintensities, the phenotype of their WM disease is less severe than nontransfused SS and Sβ^0^ (72), and may even result from different mechanisms. Furthermore, the mean hemoglobin F fractions among the nontransfused SS patients was 18%, suggesting good response to hydroxyurea. Our non-SCD and SCD cohorts were matched for hemoglobin level, so one could reasonably have expected a similar spectrum of disease. Nevertheless, this is a cross-sectional study of young adults, and there is a possibility that exposure to severely reduced arterial oxygen content prior to treatment irreversibly affected brain microstructure during brain development in transfusion-dependent non-SCD patients.

Additionally, Table 3 shows almost no contribution from monthly transfusions or LDH in the WMSs found in SCD patients. Possibly, the distribution of WMS in SCD and non-SCD looks substantially different due to uncontrolled confounders, such as chronic pain reported extensively as a burden for SCD patients (73, 74).

In childhood, SCD patients might have an intermittent pain phenotype. Around 50% of the cases evolve as a chronic pain syndrome in adulthood, with periods of lower and higher pain correlated with the ongoing vaso-occlusion (75). Table 2 shows a significant involvement of the orbito-frontal gyrus, which has been implicated in the modulation of chronic pain (76–78) and pain-related emotions (79). Furthermore, functional imaging studies have shown that regions like the thalamus and the parahippocampal gyrus, also depicted in Table 2, belong to the functional pain network (80, 81). In particular, the thalamus has been identified as a central region that processes pain (82). Anemia, by itself, is a robust biomarker of disease severity in SCD, so it is not surprising that hemoglobin levels correlate with FA in pain circuits.

Several research groups have also shown neurocognitive decline in patients with CA, suggesting a possible and early involvement of the brain even in the absence of overt strokes (83, 84). Many significant WMSs were in the prefrontal cortex (Figure 2), where WM abnormality has been associated with negative effects on neurocognitive function in CA patients (14, 15, 29). Specifically, Chai et al. reported that lower FA in the corpus callosum was associated with lower scores across nine neurocognitive measures. At the same time, Stotesbury et al. (14) found that white matter microstructural properties were associated with processing speed, where FA was the strongest predictor. Additional work in our laboratory has previously demonstrated that lower WM volume predicted low matrix reasoning scores, a measure of executive function, in CA patients and identified changes in resting-state fMRI activity in the orbitofrontal and subcallosal gyri (11). Altogether, microstructural injury patterns indicated in CA patients driven by low Hb levels may have cognitive consequences.

### Limitations

The small sample size limited our study. All participants were part of a larger project on Sickle Cell Disease at Children's Hospital Los Angeles that involved various MRI protocols (75 minutes of total scan time) and was not limited to the dMRI sequence (4 minutes). Our current approach allowed us to include all WM tracks, but it required to have high-quality structural and diffusion data, limiting us to a smaller sample size than described in Chai et al. (29). While significant sex differences have been previously indicated in the study of CA, we could not fully resolve sex disease-specific differences. The use of data from multiple cohorts of CA, whose intrinsic pathophysiology is different, weakens statistical power in the short term but opens the possibility to differentiate and characterize the unique damage induced by individual hemoglobinopathies. In addition, the control subjects were mainly recruited from family members of SCD patients, and they do not necessarily represent the non-SCD population. Consequently, the statistical differences that we found in non-SCD patients (even when controlling for log-age and sex) could be affected by random effects.

Although our contemporaneous hematological investigations are a strength of this study, we did not have previous hemoglobin or any oxygen saturation values. The use of chronic blood transfusion therapy in some of our patients potentially represents a limitation on our findings because no single hemoglobin level characterizes the hypoxic exposure. Furthermore, SCD patients are often placed on chronic transfusions later in life (than non-SCD patients) and their current hemoglobin values do not reflect their lifetime hypoxic exposure. Chronic transfusion is also a complicated therapeutic yielding improvement in erythrocyte deformability and oxygen-carrying capacity but increased viscosity in the microcirculation can potentially worsen the blood flow and oxygen delivery (85). Given our sample size, it would be tough to accurately separate the rheologic and oxygen-carrying capacity effects of red blood cells. Furthermore, the inclusion of transfusion status in the model weakens the statistical power by adding an additional degree of freedom. In addition, we were not able to assess any additional effect of low oxygen saturation on arterial oxygen content and therefore hypoxic exposure.

The constraints associated with using single-shell diffusion images and simple tensor modeling are well documented in the literature, and urge caution to draw firm conclusions from a single tensor metric like FA. This work tried to address some of these limitations by using ERFO ODFs to correctly render crossing fibers and creating WMSs to avoid characterizing connectivity by streamlining counting. However, we recognize that the information provided by FA is limited and that other methods like diffusion kurtosis imaging and neurite orientation dispersion have proven to be more robust to some of the pitfalls of DTI and could provide a more biological explanation of our current observations.

## Conclusion

To characterize the effects of CA in white matter, mean FA along the WMSs (surface connecting two ROIs) of chronic anemic patients with sickle and non-sickle anemias were compared with healthy controls. This grouping allowed the isolation of sickle hemoglobin effects in our analysis. Both CA cohorts showed localized FA differences along the WMSs of patients compared with controls but did not show differences between them. However, non-SCD patients manifested bigger systematic FA derangements in the watershed areas that were bilateral and spatially symmetrical. These results suggested that the broad spectrum of variability in disease expression in our sickle cell anemia cohort and uncontrolled confounders of mesostructure integrity affected our ability to detect widespread WM abnormalities as proposed in the literature. Nevertheless, finding interhemispheric WMSs affected in SCD aligns with previous literature reports showing decreased FA in the corpus callosum in CA patients. Recognizing both the differences and the similitudes between CA patients and the affliction that anemia causes in white matter may help develop earlier and more generalized interventions to help overcome the anemia burden.

## Data availability statement

The datasets presented in this article are not readily available because the complete data set from this trial will be made available upon reasonable request and with an approved data sharing agreement. Requests to access the datasets should be directed to JW, JWood@chla.usc.edu.

## Ethics statement

The studies involving human participants were reviewed and approved by the Institutional Review Board at Children's Hospital Los Angeles in the United States. Written informed consent to participate in this study was provided by the participants' legal guardian/next of kin.

## Author contributions

CG-Z, RL, and JW conceived and designed the study. SC, CV, BX, JS, and JW were involved in data acquisition and administrative duties. CG-Z, SC, CV, BX, JS, AJ, RL, and JW contributed to statistical analysis, manuscript writing, and designing figures. AJ, RL, and JW lead the first author's activities and decisions during the research. JW is the last author of this manuscript. All authors contributed to the article and approved the submitted version.

## Funding

This work was supported by National Heart, Lung, and Blood Institute (Grants 1U01-HL-117718-01, 1R01-HL136484-01A1, and 1F31NS106828). The National Center for Research (5UL1-TR000130-05) through the Clinical Translational Science Institute at Children's Hospital Los Angeles. A Research Career Development Fellowship supports Chau Vu from Saban Research Institute at Children's Hospital Los Angeles. Additionally, Philips Healthcare provided support for protocol development and applications engineering on a support-in-kind basis.

## Conflict of interest

Author JW is a Consultant for BluebirdBio, Celgene, Apopharma, WorldcareClinical, and BiomeInformatics. The remaining authors declare that the research was conducted in the absence of any commercial or financial relationships that could be construed as a potential conflict of interest.

## Publisher's note

All claims expressed in this article are solely those of the authors and do not necessarily represent those of their affiliated organizations, or those of the publisher, the editors and the reviewers. Any product that may be evaluated in this article, or claim that may be made by its manufacturer, is not guaranteed or endorsed by the publisher.
